# Advances in Cell-Mediated Drug Delivery for Dermatologic Diseases: Mechanisms and Current Applications

**DOI:** 10.3390/pharmaceutics17111438

**Published:** 2025-11-07

**Authors:** Lara Shqair, Iyla Draw, Tala Maya, Christopher G. Bunick, Hossein Akbarialiabad, Todd Schlesinger, Giovanni Damiani, Mahmoud Ghannoum, Ayman Grada

**Affiliations:** 1 Department of Dermatology, Icahn School of Medicine at Mount Sinai, New York, NY 10029, USA; lara.shqair@icahn.mssm.edu; 2 Department of Dermatology, University of Louisville School of Medicine, Louisville, KY 40202, USA; i0draw01@louisville.edu (I.D.); t0maya01@louisville.edu (T.M.); 3 Department of Dermatology, Yale School of Medicine, New Haven, CT 06511, USA; christopher.bunick@yale.edu; 4 Department of Dermatology, University of Utah, Salt Lake City, UT 84103, USA; hosseinakbari7575@gmail.com; 5 Department of Dermatology, The George Washington University School of Medicine and Health Sciences, Washington, DC 20037, USA; skindoc@dermandlaser.com; 6 Department of Dermatology, University of Milan, 20122 Milan, Italy; dr.giovanni.damiani@gmail.com; 7 Department of Dermatology, Case Western Reserve University School of Medicine, Cleveland, OH 44106, USA; mag2@case.edu

**Keywords:** cell-mediated drug delivery, dermatological drug delivery, targeted drug delivery, skin, tissue, skin conditions, targeted, cells, epidermis, cutaneous

## Abstract

Cell-mediated drug delivery systems represent a promising frontier in dermatologic therapy by offering enhanced targeting precision, prolonged drug release, and reduced systemic toxicity. These systems leverage the intrinsic properties of immune cells, stem cells, and skin-resident cells to migrate toward inflamed or diseased skin and deliver therapeutic agents in a controlled and biocompatible manner. This review explores the mechanistic foundations of cell-mediated delivery, including chemotaxis, phagocytosis, and immune modulation, and examines current applications in inflammatory skin diseases such as atopic dermatitis and psoriasis, cutaneous malignancies such as melanoma and cutaneous T-cell lymphoma, and chronic wound healing. Engineering approaches such as cell surface modification, exosome loading, and integration with gene editing technologies are also discussed. Finally, we highlight translational challenges related to immunogenicity, manufacturing scalability, and regulatory considerations, and propose future directions for clinical adoption in dermatology. This review provides a comprehensive overview of the current landscape and outlines the potential for cell-based delivery systems to transform the treatment of chronic and refractory skin diseases.

## 1. Introduction

Small-molecule drugs frequently face considerable challenges, including a lack of specificity for target tissues, rapid clearance from the body, and a high potential for side effects [1]. This is especially true for chemotherapeutic agents, which tend to be highly toxic [2]. Over the past few decades, drug delivery systems have surfaced as a leading strategy to address these issues by improving the safety and precision of restorative, diagnostic, and preventive agents [2]. These delivery platforms serve important roles such as extending the half-life of drugs and directing them more effectively towards their intended targets, thereby reducing exposure to healthy tissues. Despite these advances, traditional DDS methods still fall short of achieving truly personalized and highly targeted treatments [3]. This limitation presents a significant challenge to meeting the evolving demands of modern medicine, where specificity and minimizing off-target effects are necessary.

Dermatology provides a clear example of these therapeutic challenges. Biologic agents have markedly improved outcomes in diseases such as psoriasis and atopic dermatitis, yet their high cost and potential for systemic immunosuppression restrict broader adoption. Topical therapies continue to face poor adherence, and intralesional injections, while effective for localized lesions, are not feasible in patients with extensive disease. Therefore, these limitations suggest there is a need for delivery approaches that are both targeted and broadly applicable in the management of skin disorders.

In recent years, cell-mediated drug delivery systems (CMDDS) have gained substantial attention as a favorable solution to overcome these barriers [4]. By harnessing the remarkable properties of living cells, such as their ability to circulate in the bloodstream for extended periods, express diverse surface ligands, navigate complex biological environments, and respond to cellular signaling, these systems can carry and release restorative agents precisely at disease sites. This approach not only prolongs drug circulation time but also reduces cellular and tissue toxicity, making it an innovative strategy for treating a wide range of conditions.

To the best of our knowledge, this is the first comprehensive review to contextualize CMDDS within dermatology, integrating mechanistic insights, nanotechnology and bioengineering advances, and translational examples across inflammatory, pigmentary, oncologic, and regenerative skin diseases. In doing so, this work bridges the gap between basic cell-based delivery research and its dermatologic applications, while also addressing the economic and regulatory factors shaping clinical translation.

CMDDSs thus represent a new frontier in precision dermatology, enabling targeted, sustained, and adaptive delivery through living and cell-derived carriers. In this review, we explore the diverse cell types used as therapeutic vehicles, summarize current engineering strategies for constructing cell-mediated delivery systems, and discuss future directions for advancing this emerging field.

## 2. Method

This work was conducted as a narrative review to synthesize current knowledge on CMDDS, with a particular emphasis on dermatologic applications. A comprehensive literature search was performed in PubMed, Scopus, and Embase for English-language publications published between January 2000 and March 2025. The search strategy combined terms related to drug delivery, cell carriers, and dermatologic disease. Keywords included “cell-mediated drug delivery,” “cell-based delivery,” “cell carriers,” “drug delivery systems,” “mesenchymal stem cells,” “macrophage-mediated delivery,” “neutrophil-mediated delivery,” “dendritic cell vaccines,” “tumor-infiltrating lymphocytes,” “CAR-T cells,” “dermatology,” “skin disease,” “inflammatory skin disease,” “acne,” “psoriasis,” “eczema,” “atopic dermatitis,” “hidradenitis suppurativa,” “vitiligo,” “melanoma,” “cutaneous T-cell lymphoma,” “wound healing,” and “scar.” Boolean operators (AND/OR) were used to refine searches. Reference lists of relevant articles and prior reviews were also screened manually to identify additional studies.

Both preclinical and clinical studies were eligible for inclusion, along with major review articles that contributed mechanistic or translational insights. Articles were selected if they addressed (i) fundamental biological mechanisms of CMDDS, (ii) pharmacokinetic or pharmacodynamic considerations, (iii) tracking and monitoring approaches, or (iv) applications in inflammatory, autoimmune, infectious, malignant, or wound-healing dermatologic conditions.

Given the narrative design, data were synthesized qualitatively rather than quantitatively. Findings were grouped thematically into (1) biological mechanisms of CMDDS, (2) pharmacokinetics and pharmacodynamics, (3) tracking and monitoring strategies, and (4) dermatologic applications across disease categories.

## 3. The Skin

The skin, as the largest organ of the human body, serves as a vital protective barrier between the internal environment and the external world. Structurally, it is composed of three primary layers: the epidermis, dermis, and hypodermis [5,6]. The epidermis, the outermost layer, consists mainly of stratified keratinocytes that form a resilient and largely impermeable barrier designed to prevent pathogen invasion and water loss. This layer includes the highly specialized stratum corneum, composed of dead keratinized cells, which represents the main obstacle for transdermal drug penetration [5,6]. Below the epidermis lies the dermis, a thicker connective tissue layer containing blood vessels, lymphatics, nerve endings, and an assortment of immune cells such as macrophages and dendritic cells. The dermis provides structural support and mediates immune responses and tissue repair [7]. The deepest layer, the hypodermis, is composed predominantly of adipose tissue, contributing to insulation, cushion, and energy storage. Together, these layers establish a complex, dynamic environment that both protects and interacts with external and internal stimuli.

Understanding the skin’s layered architecture and cellular composition is important when considering drug delivery strategies, particularly innovative systems like CMDD. Each skin layer hosts distinct cell populations with unique roles; for example, Langerhans cells in the epidermis and macrophages in the dermis act as immune sentinels, while stem and progenitor cells contribute to regeneration [8]. These residents and circulating cells can be exploited by cell-mediated drug delivery systems to overcome the natural barrier properties of the skin and deliver therapeutics directly and selectively to diseased or inflamed sites [8].

## 4. Skin Disease and Traditional Routes of Drug Delivery

Dermatological conditions encompass a wide and diverse spectrum of disorders, including immune-mediated chronic inflammatory diseases such as atopic dermatitis, psoriasis, and hidradenitis suppurativa; infectious conditions such as cellulitis and fungal infections; autoimmune disorders, including alopecia areata and vitiligo; chronic cutaneous wounds such as diabetic ulcers and pressure sores; as well as various benign and malignant tumors such as basal cell carcinoma, squamous cell carcinoma, and melanoma [9,10,11]. Each of these conditions affects the skin’s structure and function in distinct ways, which in turn presents unique challenges for treatment. Common inflammatory conditions include atopic dermatitis, psoriasis, and urticaria, while skin cancers like basal cell carcinoma and melanoma represent malignant growths requiring specialized management [10,11].

For many inflammatory skin diseases, topical drug delivery remains the primary approach due to its direct application and non-invasive nature [12,13]. Creams, ointments, and gels are commonly used to manage symptoms like itching, redness, and inflammation [13]. However, these treatments often struggle to penetrate beyond the skin’s outer barrier, limiting their effectiveness, especially in deeper or more severe cases [13]. Oral medications are favored for systemic involvement or when lesions are widespread, but they expose the entire body to the drug, increasing the risk of side effects and affecting patient adherence due to prolonged regimens. Injectable therapies, while useful for precisely delivering biologics, are invasive and may not be practical for long-term management or localized conditions [12,13]. These shortcomings highlight the need for active, living delivery systems such as CMDDS, which can navigate biological barriers rather than rely on passive diffusion.

Similarly, treatments for infectious skin diseases rely on topical antimicrobials or systemic antibiotics, depending on the severity and extent of infection, but issues with drug penetration and systemic toxicity persist [14]. Malignant skin conditions often require surgical excision supplemented by topical or systemic therapies; delivery of drugs that target tumor cells is challenging because skin structure can hinder drug access to malignant cells without damaging healthy tissue [15].

## 5. Burden of Disease

Skin diseases affect an enormous number of people worldwide, making the development of better drug delivery methods critically important. It is estimated that nearly five billion new cases of skin and subcutaneous diseases occur globally each year. These include a wide range of disorders, from infections and inflammatory conditions to autoimmune diseases and skin cancers, impacting around 1.8 billion individuals at any given time. This widespread prevalence shows how common and varied dermatological issues truly are [16].

Beyond their sheer numbers, skin conditions impose a significant burden on individuals and healthcare systems alike. In 2019, skin diseases contributed to over 40 million disability-adjusted life years, reflecting not only the physical discomfort and disfigurement these conditions often cause but also their social and psychological impact [17]. Regions such as South Asia experience particularly high rates of both new cases and associated mortality. These rates underscore the global urgency for improved restorative options. This trend is mirrored in parts of Sub-Saharan Africa, where factors like poverty, limited access to healthcare, and environmental conditions contribute to elevated incidence and worse outcomes for fungal and infectious skin diseases. Similarly, some areas of Latin America face rising burdens of chronic inflammatory and malignant skin conditions, compounded by healthcare disparities and delayed diagnosis [17].

## 6. Economic Burden

The economic and quality-of-life burden of skin diseases is substantial and far-reaching. In the United States alone, dermatological conditions cost the healthcare system approximately $75 billion annually, covering doctor visits, hospital stays, medications, and preventive care [18]. Beyond direct medical costs, indirect expenses such as lost workdays, reduced productivity, and long-term disability add significantly to the overall impact. For instance, patients with psoriasis commonly lose an average of 8.5 workdays per year, while up to 60% report moderate to severe impairment in daily activities and social functioning [19].

Drug delivery methods themselves significantly contribute to the economic burdens associated with managing skin diseases. Topical therapies remain the most common and generally less expensive option on a per-unit basis, often costing approximately $15 to $30 per tube; however, their frequent and long-term use in chronic conditions results in substantial cumulative costs [20]. In contrast, oral and injectable systemic treatments, including biologics prescribed for severe inflammatory skin diseases or skin cancers, can cost from $12,000 to over $70,000 annually per patient, reflecting not only the high drug price but also additional expenses related to administration and ongoing patient monitoring. These high costs restrict accessibility and patient adherence, which can exacerbate disease progression and further elevate the overall economic impact [20,21,22]. Injectable therapies also pose practical and financial barriers for patients requiring long-term management due to their invasive nature and need for healthcare resources [23].

CMDDS introduce additional layers of economic complexity. Manufacturing living cell products under Good Manufacturing Practice (GMP) conditions requires specialized facilities, workforce training, and rigorous quality control, all of which increase production costs compared to conventional therapies. Autologous CMDDS, in particular, are patient-specific and resource-intensive, often requiring individualized processing that limits scalability. While this may restrict their immediate cost-effectiveness compared to mass-produced biologics, CMDDS hold potential for long-term economic benefit if they can achieve superior durability of response, reduce relapse rates, or lower the need for lifelong systemic immunosuppression [24].

Growing automation, biobanking, and cryopreservation technologies are expected to streamline manufacturing, lower costs, and expand access through standardized production pipelines. In addition, hybrid autologous allogeneic models, where universal donor cells are engineered and stored for rapid personalization, could achieve cost parity with biologics as processes mature. Parallel innovations, such as nanocarriers and microneedle-based delivery systems, further improve drug targeting, reduce systemic toxicity, and enhance patient adherence [20,21]. Collectively, these advancements suggest that CMDDS and related technologies could simultaneously mitigate clinical and economic burdens, marking an important step toward scalable, precision-based dermatologic care.

## 7. Barriers to Traditional Drug Delivery Methods and Their Limitations

### 7.1. Topical and Transdermal Delivery

The principal barrier is the stratum corneum, a ~10–20 µm layer of corneocytes embedded in a dense lipid matrix of ceramides, cholesterol, and free fatty acids that forms a tightly packed lamellar membrane [25]. This architecture severely restricts the passage of most hydrophilic and high-molecular-weight molecules and limits flux, even for lipophilic drugs. Penetration of the stratum corneum occurs mainly through the intercellular route and modulation of tight junctions in the epidermis [26]. In disease, the stratum corneum’s properties can change in complex ways that are not uniformly permissive. For example, barrier dysfunction in atopic dermatitis can increase irritancy and variability, while hyperproliferation and scaling in psoriasis alter diffusion pathways [26]. Skin also expresses enzymes that are drug-metabolizing and transporters that can degrade or efflux actives before they reach their targets [27]. Together, these factors make topical delivery unpredictable and often insufficient for deep or widespread pathology.

Chemical enhancers, iontophoresis, microneedles, and appendageal targeting can increase flux, but each have limitations. Chemical enhancers may irritate and have a limited effect on large or hydrophilic molecules [28]. Iontophoresis enables current-controlled delivery of charged drugs yet shows complex and hard-to-predict pharmacokinetics and inconsistent clinical translation [29]. Microneedles bypass the stratum corneum but are limited by mechanical requirements, application time, and manufacturing reproducibility, and can still yield variable intradermal distribution [30]. Appendageal targeting through follicles is promising but shows patient variability in follicle density, depth, and sebum content that complicates dose control [31]. While these enhancement strategies can improve skin penetration in some cases, they do not consistently provide the reliable, long-lasting, and localized drug delivery needed for complex dermatologic diseases (Table 1).

### 7.2. Oral Systemic Delivery

Oral agents are convenient for diffuse disease but undergo gastrointestinal degradation and first-pass metabolism, leading to variable bioavailability and limited delivery to the skin [32]. Increasing doses to achieve cutaneous efficacy increases systemic exposure and toxicity, necessitating laboratory monitoring and risk mitigation [32,33]. For example, in psoriasis, long-term use of oral agents like methotrexate, acitretin, or cyclosporine is associated with frequent adverse events and organ-specific toxicities that constrain duration and dosing even when clinically effective [34]. Targeted or novel orals can lessen some risks but still lack skin selectivity, so off-target effects remain a concern (Table 1).

### 7.3. Injectable Systemic Delivery

Parenteral biologics and small molecule injectables achieve higher and more reliable exposure than topical routes but distribute systemically rather than concentrating in diseased skin. Their use often requires repeated administrations, cold-chain storage, and ongoing clinical monitoring, all of which increase cost and pose adherence challenges [35]. In addition, the broad immune modulation produced by many of these agents elevates the risk of infection and other adverse events, such as injection-site reactions, and hypersensitivity [36]. These risks can be clinically significant even when the drugs demonstrate strong therapeutic efficacy.

### 7.4. Intralesional and Local Injectable Delivery

Intralesional therapy delivers the drug directly into the target tissue, which can reduce systemic exposure and improve local concentrations. However, drug distribution within lesions is often heterogeneous, depth-limited, and dependent on operator technique, which can lead to variable outcomes [37]. This approach is also less practical for dermatologic conditions that are multifocal or widespread, as serial injections can be time-consuming, uncomfortable, and difficult for patients to tolerate [37,38]. Additionally, adverse effects such as local atrophy, dyspigmentation, and procedural pain restrict its use in certain clinical scenarios [39] (Table 1). These limitations make intralesional delivery unsuitable as a consistent or scalable solution for many complex dermatologic diseases.

### 7.5. Skin Biochemistry and Immune Surveillance as Cross-Cutting Barriers

Even when drugs cross the physical barrier, the epidermis and dermis present as additional hurdles. Cutaneous cytochrome P450s, esterases, dehydrogenases, and phase II enzymes can metabolize xenobiotics, while efflux transporters can lower intracellular levels [40,41]. Epidermal immune sentinels, which are specialized immune cells within the skin that act as the first line of defense, include Langerhans cells and keratinocytes. These cells rapidly sense disturbances and can drive inflammation, irritation, or clearance responses that alter local pharmacokinetics and pharmacodynamics [42]. These properties contribute to variability between healthy and diseased skin, making it more difficult to establish predictable dose–response relationships for conventional delivery routes.

### 7.6. Implication for Therapy Design

**Figure 1:**
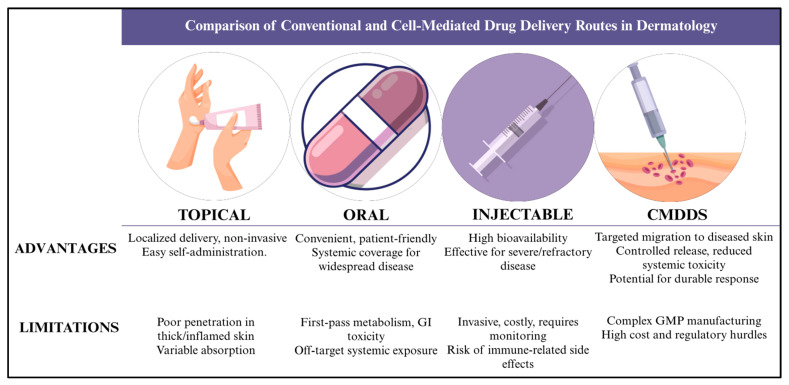
Comparison of conventional and cell-mediated drug delivery routes in dermatology.

Traditional topical, transdermal, oral, and injectable routes often face significant penetration barriers or lack tissue selectivity once in circulation. As a result, many dermatologic drugs do not reach or maintain therapeutic concentrations within inflamed or malignant skin without causing excessive systemic exposure. These shortcomings highlight the need for alternative strategies. CMDDs offer a promising solution by using living cells with innate homing, transmigration, and immunomodulatory capabilities, offering a promising solution by delivering therapeutic agents directly into diseased microenvironments while minimizing exposure to healthy tissue (Figure 1).

## 8. Development of Cell-Mediated Drug Delivery and Their Evolution

CMDDSs have progressed from conceptual models to sophisticated therapeutic platforms through advances in cell biology, bioengineering, and pharmaceutical science. Early work focused on using the intrinsic homing properties of immune cells, such as macrophages and lymphocytes, to transport therapeutic agents into sites of inflammation or tumor growth [43]. These initial approaches often relied on passive drug loading into carrier cells ex vivo, followed by reinfusion into the patient. Although early experimental studies demonstrated feasibility, limitations in drug loading efficiency, release kinetics, and carrier cell survival restricted their clinical potential [43].

### 8.1. Integration of Nanotechnology and Advances in Genetic Engineering

The field advanced significantly with the integration of nanotechnology. This enabled precise encapsulation of small molecules, proteins, and nucleic acids into nanoparticles, liposomes, and polymeric carriers that could be internalized by or attached to cell surfaces [44]. Nano-diagnostics and nanocarriers enable site-specific, controlled therapeutic delivery using systems such as liposomes, micelles, and dendrimers, illustrating how nanotechnology has transformed drug delivery strategies [45]. At the same time, advances in molecular biology broadened the capabilities of CMDDS through genetic engineering. Genetic engineering has enabled carrier cells to produce therapeutic proteins, modulate local immune responses, or express surface ligands that improve targeting to diseased tissue [46].

Recent advances have expanded CMDDS design through biomimetic nanoparticle systems that merge the targeting versatility of immune or blood cells with the stability of synthetic nanocarriers. In psoriasis and atopic dermatitis models, leukocyte-mimicking nanoparticles, such as neutrophil and macrophage-membrane-coated particles carrying methotrexate or tacrolimus, have shown selective homing to inflamed skin, enhanced drug retention, and reduced systemic toxicity [47,48]. Similarly, platelet- and RBC–membrane-coated nanoparticles exploit the natural tropism of circulating cells for sites of vascular injury, infection, or tumor growth. In preclinical models of melanoma and infected wounds, these membrane-camouflaged nanoparticles achieved preferential accumulation at damaged or inflamed tissues while delivering chemotherapeutic, photothermal, or antimicrobial payloads [47,48]. Collectively, these biomimetic platforms exemplify the next stage of CMDDS evolution, using endogenous cell membranes to confer natural adhesion, immune evasion, and inflammation-tropism while maintaining precise control over drug loading and release.

Progress in nanomaterials engineering has also enabled hybrid designs that combine synthetic components with fully living immune cells. A notable innovation involves the creation of “backpack” cargos on macrophages, in which microscopic, drug-loaded polymer disks are attached to the cell surface rather than internalized. These inflammation-homing macrophages migrate to diseased or infected tissue while continuously releasing anti-inflammatory or antimicrobial agents from their attached depots. Preclinical models of soft-tissue inflammation have demonstrated sustained local drug release and minimal off-target exposure, highlighting the feasibility of such hybrid living–synthetic CMDDS for controlled therapy within complex cutaneous microenvironments [49,50].

### 8.2. Expanding Cell Sources and Acellular Derivatives

Advances in cell sourcing have significantly broadened the scope of CMDDS, extending beyond autologous immune cells to include fibroblasts, keratinocytes, mesenchymal stem cells, and induced pluripotent stem cell (iPSC)-derived lineages. These diverse sources allow carrier cells to be harvested, genetically modified, or differentiated ex vivo to achieve disease-specific functionality, scalability, and immunologic compatibility. Such versatility enables CMDDS to be customized for a range of dermatologic applications, from inflammatory and autoimmune diseases to chronic wounds and regenerative repair.

Initially developed for oncology, CMDDS platforms have now expanded into chronic inflammatory, autoimmune, and regenerative skin conditions, supported by innovations in autologous, allogeneic, and iPSC-derived carriers [47]. Autologous cells minimize immune rejection but are resource-intensive, while allogeneic “off-the-shelf” lines enable broader accessibility and manufacturing scalability. IPSCs have emerged as a renewable cell source, capable of differentiating into keratinocytes, fibroblasts, or immune cells for dermatologic use.

More recently, attention has shifted toward acellular derivatives such as extracellular vesicles and exosomes, which preserve the signaling, targeting, and communication properties of their parent cells while avoiding the logistical and regulatory challenges associated with live-cell therapies [51]. These vesicles can encapsulate therapeutic molecules, such as microRNAs, siRNAs, proteins, or lipids, and naturally home to inflamed or damaged tissues, making them ideal for cell-free drug delivery. In dermatology, MSC-derived exosomes and EVs have demonstrated preclinical efficacy in atopic dermatitis, psoriasis, and wound healing, showing their promise as safer, more stable alternatives to whole-cell CMDDS.

### 8.3. Current Trends and Future Refinements

Current research focuses on refining targeting specificity, standardizing large-scale manufacturing, and incorporating advanced biomaterials to extend cell viability and function in vivo. Emerging trends also include the use of artificial intelligence to predict carrier cell behavior, optimize drug–cell combinations, and streamline production for clinical translation [52,53]. This progressive refinement from simple drug–cell combinations to multifunctional, bioengineered systems reflects a clear trajectory toward more precise and adaptable therapies for complex dermatologic diseases. Building on these developments, the next section examines the specific in vivo cell types currently used in CMDDS, their biological functions, and their relevance to targeted dermatologic therapy.

### 8.4. 3D Bioprinting and Tissue Engineering in CMDDS

3D bioprinting enables the precise spatial deposition of living cells, biomaterials, and growth factors to recreate skin architecture and establish controlled therapeutic release zones [54]. Bioprinted constructs that integrate keratinocytes, fibroblasts, endothelial cells, and hydrogel scaffolds can function as living depots for therapeutic secretion or controlled drug diffusion [55]. Recent platforms have integrated mesenchymal stem cells (MSCs) and nanoparticle-laden bioinks to enhance vascularization, cell survival, and payload delivery, transforming bioprinted skin equivalents into advanced CMDDS candidates [56]. As printing resolution, bioink formulation, and vascular integration improve, 3D bioprinting is poised to become a personalized manufacturing route for dermatologic CMDDS with complex geometry, lesion-specific architecture, and patient-matched design.

## 9. In Vivo Cells Used for Cell-Mediated Drug Deliveries

The human body contains a multitude of cell types, each providing distinct physiological capabilities, including pathogen recognition and clearance, tissue regeneration, and recruitment to sites of inflammation. Circulating cells represent promising vectors for therapeutic delivery due to their intrinsic ability to home to sites of inflammation, injury, or tumors, their capacity to cross biological barriers, and their low immunogenicity. The use of immune cells as carriers for therapeutic agents allows for site-specific drug delivery, capitalizing on their natural homing capabilities to pathological tissues while simultaneously minimizing off-target effects and systemic toxicity.

### 9.1. Macrophages

Immune cells are used for cell-mediated drug delivery by recruitment to diseased tissues through chemotaxis, adhesion, and transmigration in response to local chemokines and cytokines. Macrophages are highly adaptable innate immune cells originating from embryonic precursors and bone marrow. They perform essential roles in immune surveillance and homeostasis, including pathogen clearance via phagocytosis, antigen presentation to T cells, modulation of inflammatory responses, and coordination of tissue repair processes. Macrophages are particularly attractive in cell-mediated drug delivery as they offer versatile and biologically compatible platforms for targeted therapeutic transport [57]. Their intrinsic tropism for diseased or inflamed tissues, guided by chemotactic cues such as CCL2 and CXCL12, enables them to efficiently home to pathological sites [57,58]. Their capacity to transmigrate across endothelial barriers, and in some cases the blood–brain barrier, broadens their therapeutic potential across organ systems. As endogenous immune cells, macrophages display low immunogenicity and are well tolerated in circulation, enabling extended systemic half-life and reduced clearance compared to synthetic delivery systems (Table 2).

### 9.2. Neutrophils

Neutrophils are the most abundant circulating leukocytes and serve as the first line of defense in the innate immune system. They are quickly recruited to sites of infection, injury, or inflammation in response to chemotactic signals such as leukotriene B4 (LTB4), interleukin-8 (IL-8), and complement component C5a [59]. Once activated, neutrophils function as critical effectors of host defense by deploying antimicrobial defenses, including the generation of reactive oxygen species (ROS) and the formation of neutrophil extracellular traps (NETs) [60]. They function as active therapeutic agent carriers across endothelial barriers with their ability to navigate toward inflamed or diseased tissues. Neutrophils exhibit the ability to internalize nanoparticles and liposomes without significant alteration of their migratory function, which supports their potential as effective vehicles for intracellular therapeutic delivery (Table 2).

### 9.3. Dendritic Cells

Dendritic cells (DCs) are professional antigen-presenting cells (APCs) that function as central regulators between the innate and adaptive immune systems. They originate from bone marrow and are found throughout lymphoid organs and peripheral tissues, where they continuously sample the microenvironment for pathogens. Their main functions include antigen uptake, processing, and presentation to naive T cells [61]. These cells are distinguished from macrophages and neutrophils in their ability to prime naive T cells, preserving antigenic peptides for presentation on MHC molecules. This property allows DCs to efficiently initiate adaptive immune responses and induce immunological memory [62]. Given their specialized role in antigen presentation and T cell activation, dendritic cells offer distinct advantages in immunotherapeutic contexts. Strategies include loading DCs ex vivo with antigens, drugs, or nanoparticles, or using dendritic-cell-derived exosomes as delivery vehicles [62,63] (Table 2).

Building on these immunologic capabilities, dendritic-cell-targeted skin vaccination has emerged as a practical CMDDS strategy for cutaneous immunization and oncology. Microneedle-based or nanoparticle-encapsulated vaccine platforms deliver antigens or nucleic acids directly to skin-resident antigen-presenting cells, including Langerhans cells and dermal dendritic cells. Preclinical and early clinical studies have demonstrated robust antigen-specific T-cell activation and protective immune responses against melanoma and viral antigens following intradermal administration [64,65,66,67]. These findings illustrate the potential of targeting dendritic cells within the skin microenvironment to achieve localized, potent, and durable immunotherapy.

### 9.4. Mesenchymal Stem Cells

Mesenchymal stem cells are multipotent, exhibit low immunogenicity, and possess robust homing abilities to sites of injury or inflammation. Their therapeutic effects are mediated by their paracrine activity, the secretion of a complex secretome containing cytokines, chemokines, growth factors, and extracellular vesicles. The secretome modulates the local environment, promoting tissue repair, angiogenesis, anti-fibrotic effects and immunomodulation. Immunomodulatory properties of MSCs involve suppression of both innate and adaptive immune responses. They inhibit T cell proliferation, modulate dendritic cell maturation, promote regulatory T cell induction, and shift macrophage polarization toward an anti-inflammatory phenotype [68,69]. Clinical studies of MSC therapy in dermatology, including phase 1/2 trials for atopic dermatitis, psoriasis, and wound healing, have demonstrated favorable safety and early efficacy signals through their immunomodulatory secretome and anti-inflammatory cytokine release [70,71,72]. MSCs are more established clinically than iPSCs and have a well-characterized safety profile (Table 2).

Building on their parent cells’ immunomodulatory effects, MSC-derived extracellular vesicles have shown promising activity in dermatologic conditions such as atopic dermatitis and chronic wounds. These acellular vesicles carry therapeutic miRNAs, siRNAs, proteins, and lipids that reproduce many of the anti-inflammatory and regenerative effects of MSCs while avoiding the regulatory complexity of live-cell products. Preclinical and early phase 1 studies have shown reductions in inflammation, normalization of keratinocyte differentiation, and enhanced wound repair following topical or intradermal administration [73,74,75].

### 9.5. Induced Pluripotent Stem Cells

Induced pluripotent stem cells (iPSCs) are somatic cells reprogrammed to a pluripotent state, allowing them to differentiate into virtually any cell type. They exert their therapeutic effects not only through direct cell replacement but also through paracrine mechanisms. These include the secretion of extracellular vesicles, such as exosomes, which carry proteins, lipids, and nucleic acids that modulate the local tissue environment, promote regeneration, and influence immune responses [76]. They exhibit immunomodulatory effects on both innate and adaptive immune pathways. iPSC-derived cells can be engineered to generate immune cell subsets (e.g., T cells, NK cells, dendritic cells) for adoptive immunotherapy, and their secretome can modulate inflammation and promote immune tolerance [77,78]. iPSCs offer advantages as they can be generated from patient-specific cells, reducing the risk of immune rejection (Table 2). They can be expanded indefinitely in vitro, which provides a scalable source for therapeutic applications.

### 9.6. Keratinocytes

Principal skin-resident cells are leveraged in cell-mediated drug delivery strategies, particularly for gene delivery and local secretion of therapeutic proteins. Keratinocytes form most of the epidermis and can be efficiently expanded, genetically modified, and grafted onto patients. When transduced with therapeutic genes, keratinocytes can secrete bioactive proteins locally or systemically [79]. In cell-mediated drug delivery, they can be engineered to express and secrete therapeutic proteins. After grafting onto the skin, they act as a bioreactor, continuously producing and releasing the therapeutic protein. The use of keratinocyte-specific promoters allows targeted and sustained transgene expression. This approach has been shown to achieve stable secretion of proteins into the bloodstream [79,80]. Direct intradermal injection of naked DNA can also result in transgene expression in keratinocytes, leading to local or systemic effects depending on the protein produced [81] (Table 2).

#### 9.6.1. Epidermolysis Bullosa as a Clinical Example

Autologous, gene-corrected keratinocyte sheets have provided one of the most compelling clinical proofs of concept for cell-mediated delivery in dermatology. In patients with junctional or recessive dystrophic epidermolysis bullosa, epidermal stem cells were isolated, transduced ex vivo with functional transgenes, and re-grafted to generate stable, durable epidermis expressing the corrected protein [82,83,84]. These studies suggest that keratinocytes can serve as both delivery vehicles and regenerative substrates, producing therapeutic proteins in situ while restoring skin integrity (Table 3).

#### 9.6.2. Epidermal–Dermal Composite Cell Sheets

Beyond single-layer keratinocyte constructs, epidermal–dermal composite cell sheets have been widely developed as living skin equivalents for chronic wounds, burns, and ulcer repair. These autologous grafts deliver paracrine pro-healing factors and extracellular-matrix components that accelerate re-epithelialization and tissue regeneration. Clinical use and phase 2/3 trials have shown improved wound closure and graft integration, illustrating the translational maturity of cell-based CMDDS for cutaneous regeneration [85,86]

### 9.7. Fibroblasts

Fibroblasts are the principal mesenchymal cells of the dermis and are responsive to genetic modification, allowing them to be used for local delivery of therapeutic proteins, growth factors, or cytokines. They can be efficiently transfected with plasmid DNA or other gene delivery systems, including lipid-based nanocarriers, to express therapeutic proteins or growth factors [87]. Genetically modified fibroblasts can be delivered to the skin through injection or embedded in biodegradable scaffolds, allowing for localized and sustained secretion of therapeutic agents at the site of implantation. Both keratinocytes and fibroblasts secrete extracellular vesicles that modulate local immune responses and tissue repair, further enhancing their therapeutic potential in cell-mediated drug delivery [87,88]. Autologous cultured fibroblast suspensions have progressed to clinical approval for aesthetic and regenerative dermatology. Injected fibroblasts secrete extracellular-matrix proteins and trophic growth factors that remodel dermal architecture and improve atrophic acne scars and rhytids. Phase III trials and long-term follow-up have confirmed their safety and sustained benefit, representing one of the earliest approved CMDDS applications in dermatologic practice [89,90,91,92].

## 10. Biological Mechanisms of Action of CMDDS

### 10.1. Homing and Targeted Migration to Diseased Tissue

A defining feature of many cell types used in cell-mediated drug delivery systems, including macrophages and mesenchymal stem cells, is their intrinsic ability to home to sites of inflammation, injury, or tumor growth. This migratory behavior is guided by chemokine gradients and adhesion molecule interactions that facilitate targeted trafficking to pathological microenvironments [93]. In inflammatory conditions, activated endothelial cells upregulate selectins, integrins, and immunoglobulin superfamily members (ICAM-1, VCAM-1), which mediate rolling, adhesion, and transendothelial migration of circulating cells [94]. Chemokines such as CCL2, CXCL12, and CXCL8 bind to their receptors CCR2, CXCR4, and CXCR1/2 on macrophages and MSCs, organizing directed migration along chemotactic gradients [95]. Once within the target tissue, these cells can localize with high specificity, allowing therapeutic delivery while minimizing systemic exposure.

### 10.2. Cellular Internalization of Therapeutic Payloads

A fundamental biological mechanism underlying the use of certain cell types in cell-mediated drug delivery is their intrinsic capacity for phagocytosis and endocytosis, which allows for internalization of therapeutic payloads such as nanoparticles, liposomes, and polymeric micelles [96]. Phagocytes, including macrophages, neutrophils, and dendritic cells, are highly efficient at internalizing engineered particulate carriers. This process is mediated by a variety of receptor–ligand interactions, involving Fc receptors, complement receptors, scavenger receptors, and pattern recognition receptors such as Toll-like receptors (TLRs), which enable the recognition and binding of opsonized drug carriers [97]. Once internalized, therapeutic payloads are compartmentalized within endocytic vesicles that traffic through the endo-lysosomal pathway. Environmental cues such as acidic pH, lysosomal enzymes, or redox gradients can be strategically used to trigger controlled release.

### 10.3. Payload Release via Secretion and Cell Fusion

Many cell types used in cell-mediated drug delivery systems can release their therapeutic payloads through endogenous secretion pathways or direct membrane fusion with target cells. These release mechanisms exploit the cells’ native communication strategies, allowing for controlled drug transfer [98]. One mechanism is the secretion of extracellular vesicles, including exosomes and micro-vesicles, which are naturally enriched in proteins, lipids, and nucleic acids [99]. Mesenchymal stem cells or dendritic cells can incorporate therapeutic agents into EVs during biogenesis, enabling the delivery of bioactive molecules to recipient cells via endocytosis or membrane fusion [99,100]. This vesicle-mediated transport offers several advantages, including stability in circulation, protection of cargo from degradation, and intrinsic tissue-targeting capabilities dictated by vesicular surface proteins. Another mechanism involved direct fusion between the drug carrier cells and their target. Certain immune cells, such as macrophages, can form transient fusion events to facilitate the transfer of cytoplasmic contents directly into target cells [101]. Programmed cell death or activation-induced lysis can also result in controlled drug release [102]. While this approach can enhance local drug exposure, it must be carefully balanced to avoid premature release during systemic circulation.

### 10.4. Immunomodulatory Effects at Target Sites

Certain cell types used in cell-mediated drug delivery systems can actively reshape the immune environment at the target site. Mesenchymal stem cells exert broad-spectrum immunoregulatory effects through both contact-dependent mechanisms and soluble mediators, including transforming growth factor-β (TGF-β), interleukin-10 (IL-10), prostaglandin E2 (PGE2), and indoleamine 2,3,0-dioxygenase (IDO) [103]. These factors suppress the activation and proliferation of effector T cells, inhibit dendritic cell maturation, and promote the expansion of Tregs, fostering an anti-inflammatory environment [104]. Regulatory T cells act as direct enforcers of immune tolerance by suppressing effector T cell responses and limiting the activity of antigen-presenting cells via cytotoxic T lymphocyte antigen 4 (CTLA-4) mediated signaling and IL-2 consumption [105]. They can be engineered to release anti-inflammatory cytokines or exert cell-to-cell contact-dependent suppression.

### 10.5. Pharmacokinetics and Pharmacodynamics in Dermatologic CMDDS

CMDDSs alter the pharmacokinetic and pharmacodynamic profiles of therapeutics by actively guiding them to diseased skin and protecting them from premature clearance. After administration, CMDDSs use their intrinsic migratory capacity to cross endothelial barriers and enter inflamed or malignant skin, improving the drug absorption compared with passive diffusion [106]. Once in the circulation, these carrier cells prolong drug half-life by protecting therapeutic agents inside the cell or on its surface [107]. This delays systemic clearance and concentrates distribution at the disease site.

Within the local microenvironment, carrier cells can protect drugs from enzymatic breakdown. They can also enable controlled release through mechanisms such as extracellular vesicle secretion, gradual cell degradation, or activation by environmental cues including pH, protease activity, or redox state [51]. CMDDS and their released therapeutics are then cleared through the reticuloendothelial system, hepatic metabolism, renal excretion, and immune-mediated removal of apoptotic carrier cells [108]. The way CMDDSs move drugs through the body and control their breakdown can improve treatment effectiveness for chronic skin diseases. These features also emphasize the need for in vitro engineering methods that improve stability, loading capacity, and targeting accuracy before use in patients.

Because these systems depend on complex biodistribution and clearance dynamics, effective strategies to track CMDDS in vivo are critical. Noninvasive monitoring not only provides important insights into their migration and persistence but also guides optimization of dosing, safety, and therapeutic outcomes.

### 10.6. Tracking and Monitoring CMDDS In Vivo

An essential aspect of developing CMDDSs is the ability to noninvasively monitor their biodistribution, persistence, and functional behavior after administration. Molecular imaging approaches provide real-time insight into carrier cell trafficking and therapeutic engagement. Positron emission tomography (PET) labeling with radionuclides such as ^89Zr-oxine enables sensitive, quantitative tracking of CMDDS over extended periods, though signal dilution with cell division can limit resolution [109]. Magnetic resonance imaging (MRI) provides high spatial resolution when cells are labeled with superparamagnetic iron oxide (SPIO) nanoparticles, allowing precise anatomical localization, but suffers from lower sensitivity relative to nuclear imaging [110]. Near-infrared (NIR) fluorescent dyes represent another strategy, which offers real-time visualization of CMDDS migration in small animal models and potential clinical translation with advanced optical imaging technologies [111]. Combining multiple imaging modalities in preclinical and clinical studies enhances the accuracy of CMDDS tracking and facilitates optimization of dosing strategies, safety monitoring, and assessment of therapeutic efficacy.

## 11. In Vitro Engineering and Enhancement Methods

### 11.1. Passive Loading via Endocytosis and Phagocytosis

Passive loading relies on the cell’s natural endocytic or phagocytic pathways to internalize therapeutic cargo without external physical or chemical disruption. This approach is commonly applied to macrophages and neutrophils, which can readily engulf nanoparticles, liposomes, and polymeric micelles through receptor-mediated endocytosis. For non-phagocytic cells, uptake can be facilitated by optimizing nanoparticle size and surface charge to match specific endocytic pathways [112].

### 11.2. Electroporation for Enhanced Intracellular Delivery

Electroporation employs short, high-voltage electrical pulses to permeabilize the cell membrane, enabling the direct entry of charged particles like nucleic acids, proteins, or small-molecule drugs. This technique can achieve high loading efficiency and is used for genetic modification of immune cells, including T cells and dendritic cells [113]. Optimization of voltage parameters, pulse duration, and cell handling protocols is important due to the mechanical stress and membrane disruption associated with electroporation.

### 11.3. Nanoparticle Surface Conjugation and Intracellular Loading

An emerging strategy involved attaching drug-loaded nanoparticles to the surface of carrier cells or incorporating them into the cytoplasm through controlled internalization. Surface conjugation is achieved through covalent linkages or antibody-mediated binding to cell membrane proteins [114,115,116]. By localizing the payload externally, nanoparticle backpacks preserve intracellular processes and signaling pathways, maintaining cell viability, polarization, and chemotactic responsiveness. Surface-bound nanoparticles can be engineered to release their cargo under specific environmental triggers. The intracellular loading of nanoparticles is optimized through endocytic or phagocytic pathways [117]. This method shields the therapeutic cargo from immune clearance and enzymatic degradation during systemic circulation, allowing sustained protection until the carrier cell reaches the target site.

### 11.4. Cell Surface Modification for Targeting Specificity

Cell surface modification represented an engineering strategy to enhance the targeting specificity and functional performance of cell-mediated drug delivery systems. By coating the plasma membrane with targeted ligands, antibodies, or other functional moieties, carrier cells can be programmed to recognize and bind selectively to receptors expressed in pathological tissues. Inflamed endothelial cells overexpress vascular cell adhesion molecule-1 (VCAM-1) and intercellular adhesion molecule-1 (ICAM-1), which can be targeted using antibodies, peptides, or aptamers conjugated to the carrier cell membrane [118]. Similarly, in melanoma, T cells engineered with tumor antigen-binding ligands demonstrate enhanced infiltration and cytotoxicity within tumor nodules [119]. Surface modifications must be optimized to preserve carrier cell viability, migratory capacity, and immune compatibility, as excessive or non-specific labeling may impair functional performance or trigger clearance by the host immune system.

### 11.5. Genetic Engineering of Carrier Cells

Genetic engineering is a transformative approach for enhancing the therapeutic potential of cell-mediated drug delivery systems. Researchers equip cells with capabilities that extend their native physiological repertoire by reprogramming cellular functions at the genomic level. This included on-demand drug synthesis, immune modulation, and enhanced survival in hostile environments [120]. Gene delivery can be accomplished using viral or non-viral methods. Viral methods remain the gold standard for stable, long-term expression due to their high transduction efficiency and ability to integrate transgenes into the host genome [120]. For example, lentiviral systems have been used to engineer T cells and dendritic cells to secrete interleukins, tumor necrosis factor antagonists, or checkpoint inhibitors directly within diseased tissues. Non-viral systems offer the advantages of reduced immunogenicity and lower risk of insertional mutagenesis, making them beneficial for short-term applications [121]. CRISPR, a gene-editing technology derived from a natural bacterial defense system, allows highly specific insertion, deletion, or replacement of DNA sequences, creating cells capable of secreting therapeutic proteins only under stimuli.

### 11.6. Exosome Engineering for Cell-Free Therapeutic Delivery

Exosome engineering harnesses the intrinsic communication machinery of cells without the logistical and safety challenges associated with administering live, replicating cells. Exosomes are nanoscale extracellular vesicles of endosomal origin, secreted by all cell types, and naturally enriched in proteins, lipids, and nucleic acids [122]. Their small size, lipid bilayer stability, and endogenous targeting properties enable them to traverse biological barriers, including dermal extracellular matrix and, in some cases, the blood–brain barrier [123]. Exosomes can be engineered to carry therapeutic payloads using either endogenous or exogenous loading approaches. Endogenous loading involves modifying the parental cells so that the therapeutic cargo is selectively packaged into exosomes during biogenesis [124]. Exogenous loading requires the direct incorporation of drugs, nucleic acids, or imaging agents into purified exosomes post-isolation [125]. While endogenous loading offers better control, exogenous methods allow for more flexibility in selection and higher loading efficiencies for certain therapeutics.

### 11.7. Comparative Summary of In Vitro Engineering Approaches

Among in vitro engineering methods for CMDDS, passive loading offers procedural simplicity but often yields lower loading efficiency; electroporation enables high intracellular cargo insertion but can compromise cell viability and function; surface conjugation preserves cell physiology and allows controlled release but typically limits cargo volume; and genetic engineering affords durable transgene expression and autonomous payload production but carries greater regulatory complexity and safety risk. The optimal method therefore depends on the therapeutic payload (e.g., small molecule, nucleic acid, protein), the desired release kinetics, carrier cell type, and clinical indication.

## 12. Dermatological Applications of CMDDS

**Figure 2:**
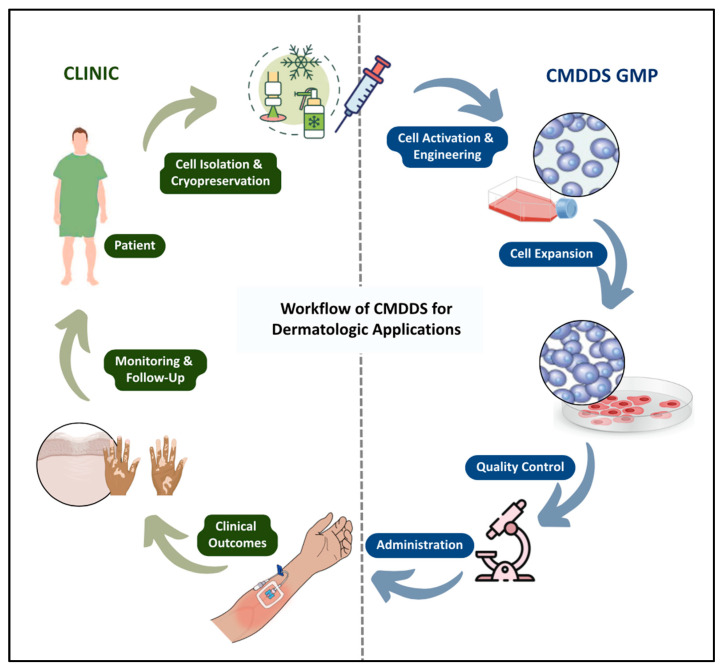
Workflow of cell-mediated drug delivery systems. Patient cells are isolated (autologous or allogeneic) and cryopreserved. Cells undergo activation and engineering (drug loading, genetic modification, or exosome enrichment), followed by large-scale GMP expansion under bioreactor conditions. Quality control ensures potency, purity, sterility, and absence of contaminants. Cells are re-administered via intravenous infusion, intradermal injection, or grafting. Clinical outcomes include lesion clearance, repigmentation in vitiligo, and wound healing.

Dermatology serves as an ideal platform for advancing cell-mediated drug delivery systems, given the accessibility of the skin and the complex therapeutic challenges it presents. Many skin disorders are chronic, relapsing, and inflammatory, arising from varied mechanisms such as immune dysregulation, barrier dysfunction, microbial imbalance, or malignant transformation. Standard treatments, including topical agents, oral medications, injectables, and surgical approaches, often have limitations, such as suboptimal tissue penetration, systemic toxicity, invasive procedures, or incomplete and temporary benefits. CMDDS represents a promising shift, harnessing living cells to home to disease sites, modulate local immune responses, and provide sustained, targeted therapy. This section examines the potential of these strategies across a range of skin conditions, including prevalent inflammatory diseases (acne, psoriasis, eczema), pigmentary disorders (vitiligo), malignancies (melanoma, cutaneous T-cell lymphoma), neutrophil-mediated conditions (hidradenitis suppurativa), and chronic wounds or scarring (Figure 2).

### 12.1. Acne Vulgaris

Acne vulgaris is a chronic inflammatory skin disorder characterized by follicular hyperkeratinization, excess sebum production, proliferation of *Cutibacterium acnes*, and a complex inflammatory cascade involving both innate and adaptive immune responses [126,127]. While conventional therapies like topical retinoids and antibiotics are effective for many patients, they are often limited by side effects and bacterial resistance.

CMDDSs provide a new means of treating disease at its source within the skin microenvironment. MSCs contain potent immunomodulatory and tissue-regenerative properties by secreting anti-inflammatory cytokines (IL-10, TGF-β) and bioactive molecules that downregulate pro-inflammatory mediators like IL-1, TNF-α, and IL-8. The cytokine profile also reduced the recruitment of neutrophils and the formation of inflammatory papules and pustules. Beyond inflammation control, MSCs have the potential to regulate sebaceous gland activity through their paracrine effects. They secrete a diverse array of bioactive molecules, including cytokines, chemokines, growth factors, and extracellular vesicles that can modulate environments and cell behavior, all of which are relevant to sebaceous gland physiology and pathology [126]. These factors also play a role in stimulating fibroblast and keratinocyte proliferation, accelerating wound healing, and improving scar quality [127].

Recent preclinical findings substantiate the translational potential of CMDDS in acne. MSC-derived exosomes loaded with anti-inflammatory microRNAs and cytokines significantly suppressed *Cutibacterium acnes* induced inflammation in murine acne models, reducing IL-1β and TNF-α expression and normalizing keratinocyte differentiation [128]. Likewise, engineered macrophages carrying clindamycin-, minocycline-, or doxycycline-loaded nanoparticles have shown improved follicular penetration and sustained antimicrobial release. Collectively, these results show that CMDDS can effectively deliver both biologic and small-molecule agents to sebaceous follicles, a key limitation of conventional topical or systemic regimens.

### 12.2. Psoriasis

MSCs interact with key immune cells implicated in psoriasis pathogenesis, including dendritic cells, T cell subsets (Th1, Th17, Tregs), neutrophils, and keratinocytes, through both direct cell contact and paracrine secretion of anti-inflammatory cytokines. These actions shift the immune system from pro-inflammatory to a regulatory state, reduce the Th17/Treg imbalance, inhibit neutrophil extracellular trap formation, and decrease keratinocyte proliferation, collectively leading to reduced inflammation and improved skin barrier function [129]. In addition to their immunoregulatory properties, MSCs secrete growth factors and extracellular vesicles that stimulate angiogenesis and tissue regeneration, further facilitating the resolution of psoriatic plaques [130].

Macrophages also play a pivotal role in amplifying psoriatic inflammation by releasing cytokines that promote Th17 activation. Accordingly, CMDDS-based therapeutic strategies are being developed to deplete pathogenic macrophages, inhibit pro-inflammatory (M1) polarization, or reprogram macrophages toward anti-inflammatory (M2) phenotypes [131].

Multiple preclinical studies have shown the translational strengths of CMDDS in psoriasis. Intradermal or intravenous administration of human MSCs in imiquimod-induced psoriatic mice markedly reduced epidermal hyperplasia and Th17-associated cytokines [132]. Exosome-based CMDDS derived from MSCs have also been engineered to carry miR-210 and IL-10, producing dose-dependent improvement in erythema and scaling in psoriatic skin [132,133].

Expanding on this concept, recent studies have developed cell-derived extracellular vesicles engineered to deliver siRNA directly to keratinocytes and dendritic cells within inflamed skin. By targeting the epithelial–immune axis, these EVs silence pro-inflammatory gene expression and restore immune homeostasis in conditions such as psoriasis and atopic dermatitis [134,135,136]. Topical or intradermal application of EV–siRNA complexes in murine models has produced significant reductions in epidermal hyperplasia, cytokine overexpression, and immune-cell infiltration, representing a promising cell-free CMDDS strategy for treating inflammatory dermatoses.

### 12.3. Eczema

MSC-derived secretome and exosomes contain growth factors and cytokines that directly increase keratinocyte proliferation, migration, and support of basal keratinocyte populations. In addition, MSC-derived exosomes induce de novo synthesis of ceramides and other barrier lipids, restoring the lamellar structure of the stratum corneum [137]. These mechanisms are particularly relevant for eczema, where impaired keratinocyte differentiation and reduced ceramide content contribute to barrier dysfunction, inflammation, and pruritus [138]. The immunomodulatory properties of MSCs attenuate Th2-driven inflammation, central to eczema pathogenesis, by downregulating cytokines such as IL-4, IL-5, and IL-13, which are also targeted by current biologic therapies like dupilumab and tralokinumab [139]. Topical application of MSC-derived exosomes restored ceramide synthesis and barrier integrity in atopic-dermatitis mouse models, highlighting translational potential for CMDDS-based barrier repair [137,138,139].

Beyond traditional cellular therapies, advances in probiotic and commensal live biotherapeutics have introduced a new dimension to CMDDS in dermatology. These approaches harness naturally occurring skin microbes, such as *Roseomonas mucosa*, *Staphylococcus hominis*, and ammonia-oxidizing bacteria, to deliver therapeutic molecules directly within the cutaneous microenvironment. By secreting antimicrobial peptides, sphingolipids, and nitric oxide, these engineered or autologous strains restore microbial balance, suppress pathogenic *Staphylococcus aureus*, and modulate skin inflammation [140]. Phase 1 and 2 clinical studies have shown improved disease severity and pruritus in atopic dermatitis, with additional trials underway exploring their efficacy in acne and other dysbiosis-driven skin disorders [141,142,143,144]. These expand the scope of CMDDS strategies to include microbiome-based therapeutics capable of in situ drug synthesis and sustained immunomodulation.

### 12.4. Melanoma

Immune cells, including T cells, macrophages, and dendritic cells, can be engineered or loaded with therapeutic agents or nanoparticles, leveraging their natural homing properties to deliver drugs within the tumor microenvironment. Among these, adoptive T-cell therapies, including tumor-infiltrating lymphocytes (TILs), CAR-T cells, and T-cell receptor (TCR) engineered products, represent the most clinically advanced CMDDS platforms for cutaneous malignancies. In melanoma, autologous TIL therapy has achieved durable tumor regression and is now FDA-approved, demonstrating how living immune cells can be harnessed to achieve precise, long-lasting antitumor immunity. Combination strategies pairing TILs or CAR-T cells with immune checkpoint inhibitors such as nivolumab and pembrolizumab have further improved durable response rates and survival outcomes in advanced melanoma [145,146,147,148,149]. Beyond melanoma, engineered T-cell strategies are under investigation for cutaneous T-cell lymphoma (CTCL), where adoptively transferred autologous T cells can recognize tumor-associated antigens and mediate selective clearance of malignant clones [149]. Together, these therapies exemplify how cell-mediated platforms can deliver potent, targeted cytotoxicity within diseased skin while minimizing off-target toxicity.

Mesenchymal stem cells can also be engineered or loaded with therapeutic agents through strategies such as genetic modification, direct loading, or surface modification. Studies have shown that MSCs engineered to secrete IL-12 or loaded with chemotherapeutics can suppress tumor growth, reduce metastasis, and enhance antitumor responses while minimizing systemic toxicity [150]. These approaches show the versatility of CMDDS in enhancing drug bioavailability, prolonging therapeutic exposure, and modulating the local tumor immune milieu.

In parallel, other “live biologic” therapies have reached the clinic. Talimogene laherparepvec (T-VEC), an oncolytic herpes simplex virus approved for melanoma, is not a CMDDS in the strict sense but illustrates how engineered biologic systems can selectively target tumor cells and stimulate local antitumor immunity [151,152,153]. Related strategies are emerging using bacteriophage- or virus-based delivery via carrier cells, in which engineered phages or oncolytic viruses deliver cytotoxic or gene-modulating payloads to malignant or infected tissues. Preclinical studies have shown effective targeting of melanoma and acne biofilms, showing potential for precise destruction of tumor cells or pathogenic bacteria while sparing healthy tissue [154,155]. Collectively, these viral and phage platforms extend CMDDS principles to non-cellular yet biologically guided delivery systems capable of selective replication and local therapeutic amplification.

### 12.5. Vitiligo

Stem cells derived from a patient’s own body are used to repopulate melanocytes and restore skin color in depigmented areas for the treatment of vitiligo by either direct transplantation of autologous melanocytes or by differentiating autologous stem cells into functional melanocytes or melanocyte precursors, which are then transplanted into depigmented skin [156]. Autologous cell suspensions containing melanocytes, keratinocytes, and fibroblasts have been shown to integrate into the epidermis, produce melanin, and restore pigmentation [157]. Additionally, MSCs may contribute to repigmentation by modulating the local immune environment and reducing oxidative stress, thereby supporting melanocyte survival and function.

Autologous melanocyte–keratinocyte transplantation (MKTP) represents one of the most clinically validated CMDDS approaches for pigmentary disorders. In this procedure, melanocytes and keratinocytes are co-cultured and re-grafted onto depigmented areas, where they integrate into the epidermis, resume melanin production, and restore pigmentation. Long-term follow-up from specialized centers demonstrates durable color match, minimal adverse effects, and sustained repigmentation, suggesting MKTP as a mature example of cell-mediated cutaneous therapy [158,159,160].

### 12.6. Hidradenitis Suppurativa

Hidradenitis Suppurativa (HS) is a persistent, relapsing inflammatory condition of the hair follicle, marked by painful nodules, abscesses, and sinus tracts that most often develop in intertriginous areas. Despite the use of antibiotics, such as clindamycin, rifampin, and doxycycline, biologic agents targeting TNF-α (e.g., adalimumab, infliximab) and IL-17 (e.g., secukinumab), and surgical interventions, many patients experience incomplete responses and frequent relapses [161]. Newer topical formulations, such as foams and gels, can enhance drug delivery for localized HS lesions by improving penetration, ensuring coverage of challenging anatomical sites, and supporting better patient adherence versus older creams or ointments [162].

CMDDS may address remaining gaps by employing neutrophil- or macrophage-based carriers to deliver anti-inflammatory or antimicrobial payloads directly to inflamed follicles and abscesses. Mesenchymal stem cells could also exert beneficial effects in HS through secretion of IL-10, TGF-β, and prostaglandin E2, reducing neutrophil infiltration and promoting tissue repair. Additionally, engineered immune cells capable of releasing targeted biologics at HS lesions could minimize systemic toxicity while improving efficacy.

### 12.7. Cutaneous T-Cell Lymphoma

CTCL encompasses a group of extranodal non-Hodgkin lymphomas characterized by malignant proliferation of skin-homing T cells, including mycosis fungoides and Sézary syndrome. Standard treatments such as phototherapy, topical steroids, systemic retinoids, and interferons offer symptomatic control but are rarely curative in advanced disease [163]. CMDDS is promising in this setting, by enabling precise immunotherapy delivery. Dendritic cell-based vaccines, pulsed with tumor antigens, have been tested in CTCL with the goal of priming robust anti-tumor T-cell responses [61]. Moreover, engineered autologous T cells, including CAR-T and TCR-modified approaches, can be adapted to recognize CTCL antigens and enhance cytotoxic clearance of malignant clones. Harnessing these strategies within CMDDS platforms could expand therapeutic options for patients with refractory or advanced CTCL while minimizing off-target immunotoxicity.

### 12.8. Wound Healing and Scars

Fibroblasts are central to wound healing and tissue regeneration by migrating into the wound bed, proliferating, and synthesizing extracellular matrix components, especially type I and III collagen, which provide structural support and facilitate re-epithelialization [164]. Additionally, they mediate wound contraction and help minimize scar formation through regulated ECM modeling. MSCs and their secretome can shift the wound environment toward regeneration rather than fibrosis, reducing scar formation and improving tissue quality [165]. They contribute to wound healing by differentiating into skin cell lineages, secreting paracrine factors that modulate inflammation, promote angiogenesis, stimulate resident cell proliferation, and enhance ECM production. Co-transplantation of MSCs with fibroblasts further augments fibroblast proliferation, migration, and ECM deposition, leading to superior outcomes in wound closure and scar minimization [166].

Complementing these cellular approaches, platelet-rich plasma has emerged as a practical, clinically adopted biologic that leverages autologous cell-derived growth factors for tissue regeneration. PRP contains high concentrations of platelet-derived growth factor (PDGF), vascular endothelial growth factor (VEGF), and TGF-β, which collectively stimulate angiogenesis, collagen synthesis, and epithelial regeneration [167,168]. Clinical studies have shown its benefit across dermatologic applications, including chronic wounds, acne scars, androgenetic alopecia, photoaging, and vitiligo [169,170,171]. By promoting localized cytokine release and tissue repair, PRP represents a simplified and cost-effective CMDDS analog with a strong safety record, though standardization across preparation methods remains variable.

In severe or treatment-refractory skin and soft-tissue infections, particularly in neutropenic or immunocompromised patients, granulocyte transfusions have been investigated as a specialized, immune cell-based therapeutic option. These transfusions deliver viable neutrophils and other phagocytic cells capable of releasing antimicrobial peptides and mounting rapid innate immune responses against fungal and bacterial pathogens [172,173,174]. While clinical outcomes have been mixed due to variability in donor cell quality, timing of administration, and host immune status, granulocyte transfusions remain an important example of CMDDS in clinical practice, reinforcing how living immune cells can restore antimicrobial defense and enhance infection control in compromised skin environments.

**Table 3:** Cell-Mediated Therapeutic Delivery in Dermatology: Modalities and Evidence. Abbreviations: EB = epidermolysis bullosa; AD = atopic dermatitis; CTCL = cutaneous T-cell lymphoma; ECM = extracellular matrix; APC = antigen-presenting cell; EV = extracellular vesicle; NP = nanoparticle; AMPs = antimicrobial peptides; NO = nitric oxide.

Cell-Based/Derived Modality	Dermatological Application	Therapeutic Payload	Development Stage	Reference
Autologous gene-corrected keratinocyte sheets	Junctional/recessive dystrophic EB (ex vivo–corrected epidermal stem cells, re-grafted)	Functional gene/protein via engrafted keratinocytes	Clinical case series; phase 3 programs	[82,83,84]
Autologous epidermal/dermal cell sheets	Chronic wounds, burns, ulcers (living skin equivalents)	Paracrine pro-healing factors; ECM	Clinical practice; phase 2/3 trials	[85,86]
Autologous fibroblast injections	Atrophic acne scars and rhytids (dermal fibroblast suspensions)	ECM proteins; trophic factors	Approved product history; niche clinical use	[89,90,91,92]
Melanocyte–keratinocyte cell transplantation (MKTP/MKCT)	Stable vitiligo repigmentation	Functional melanocytes (melanin production)	Specialized centers; long-term follow-up	[158,159,160]
Mesenchymal stromal/stem cells (MSC)	AD, psoriasis, scars, radiation injury, wounds (local or IV)	Immunomodulatory secretome; anti-inflammatory cytokines	Phase 1/2a; expanding preclinical base	[70,71,72]
MSC-derived extracellular vesicles (exosomes)	AD and chronic wounds (topical/intradermal)	miRNA/siRNA, proteins, lipids (cell-free therapy)	Preclinical to phase 1	[73,74,75]
Dendritic-cell–targeted skin vaccination (incl. microneedles)	Cutaneous immunization; melanoma/viral antigens	Antigen/genes to skin APCs (Langerhans/DCs)	Clinical vaccinology; near-clinical oncology	[64,65,66,67]
Adoptive T-cell therapies (TILs/engineered T cells)	Cutaneous melanoma; CTCL	Cell-delivered cytotoxicity to tumor	Established in melanoma; phase 2 in CTCL	[146,147,148,149]
Leukocyte-mimicking/leukocyte-hitchhiking nanoparticles	Psoriasis/AD models (neutrophil/macrophage membrane–coated NPs carrying MTX/tacrolimus)	Small-molecule immunomodulators with inflamed-skin homing	Robust preclinical; emerging translational data	[34,47]
Platelet- or RBC-membrane–coated nanoparticles	Melanoma; infected wounds (damage-site tropism)	Chemo/photothermal agents; antibiotics	Preclinical (oncology/wound focus)	[47,48]
“Backpack” cargo on living immune cells	Inflammation-homing macrophages carrying drug-loaded micro-disks	Localized anti-inflammatories/antibiotics	Preclinical proofs-of-concept	[49,50]
Probiotic/commensal live biotherapeutics	AD and acne (Roseomonas mucosa, Staph. hominis, ammonia-oxidizers)	In situ AMPs, sphingolipids, nitric oxide	Phase 1/2 signals; ongoing trials	[141,142,143,144]
Engineered skin microbes	Designed Staph. epidermidis secreting therapeutic proteins (antipruritics, AMPs)	On-skin biologics/peptides	Preclinical; first-in-human pathways	[140]
Bacteriophage/oncolytic virus via cells	Intratumoral melanoma therapy; phage for acne biofilms	Genes/cytotoxic payloads to target cells	Clinical (oncolytic viruses); preclinical (phage)	[152,153,154,155]
Cell-derived vesicles targeting keratinocytes/DCs (siRNA)	Psoriasis/AD models (EV-siRNA to epithelial–immune axis)	Nucleic acids (gene silencing)	Preclinical dermatology	[134,135,136]
Platelet-rich plasma (PRP)	Chronic wounds, acne scars, androgenetic alopecia, photoaging, vitiligo	Growth factors (e.g., PDGF, VEGF, TGF-β), cytokines for regeneration and anti-inflammation	Clinical practice; multiple trials with variable standardization	[169,170,171]
Granulocyte transfusions	Severe skin/soft tissue infections in neutropenic patients (e.g., fungal, bacterial)	Antimicrobial peptides, phagocytic cells for immune response	Specialized clinical use; mixed efficacy in trials	[172,173,174]

## 13. Challenges in Translating CMDDS to Clinical Practice

### 13.1. Autoimmune Complications

The risk that the patient’s immune system may reject or attack carrier cells and create immune-related complications is a significant translational challenge for cell-mediated drug delivery systems. While MSCs have low immunogenicity due to low expression of MHC class II and costimulatory molecules, they are not fully immuno-privileged. Allogeneic MSCs can be recognized and eliminated by host immune responses, especially after repeated administration, leading to reduced therapeutic efficacy and potential for immune-mediated adverse events such as alloimmunization, inflammation, or even graft-versus-host-like reactions [175]. Immune cell-based carriers are also susceptible to immune rejection, especially if they are genetically modified, which can trigger host-versus-graft responses or off-target activation [176]. Inflammatory environments, as seen in skin cancers, vitiligo, and wounds, may further enhance immune recognition and clearance of exogenous cells. Strategies to mitigate these risks include autologous cells, transient immunosuppression, genetic engineering to reduce immunogenicity, and encapsulation or cell membrane modification to evade immune detection.

### 13.2. Complications of Production of Specialized Cells

Large scale expansion of cells, such as fibroblasts, MSCs, and immune cells, often leads to cellular senescence, loss of potency, and altered phenotype. Extended culture, especially in suboptimal or non-physiological conditions, can reduce proliferation, differentiation capacity, and therapeutic efficacy [177]. Ensuring that each batch of cells performs consistently is complicated by donor variability, differences in tissue source, and subtle changes in culture conditions, all of which can affect cell phenotype, secretome, and therapeutic efficacy [178]. Rigorous quality control and standardization are required to minimize these sources of variability.

### 13.3. Regulatory Requirement Restraints

Complex regulatory requirements from agencies such as the United States FDA and the European Medicines Agency can significantly slow the approval and clinical availability of new cell-mediated drug delivery systems due to rigorous oversight of manufacturing, quality control, and demonstration of consistent safety and efficacy. These therapies are highly individualized, often involving living cells with complex biological properties, which makes standardization and reproducibility challenging [179]. Regulatory agencies require detailed characterization of the cell product, including identity, purity, potency, and stability as well as robust evidence of manufacturing consistency and control of potential contaminants or adventitious agents [180]. Batch-to-batch variability, donor heterogeneity, and the inherent complexity of living cell products increase the burden of quality control.

Regulatory requirements also differ between autologous and allogeneic products. Autologous CMDDS, particularly when minimally manipulated and re-administered to the same patient, may follow less stringent frameworks in the U.S. (21 CFR Part 1271) or exemptions under EMA guidance, expediting clinical translation. In contrast, allogeneic products are generally regulated as biologics or ATMPs, requiring comprehensive Investigational New Drug (IND) applications, phase-based clinical trials, and Biologics License Applications (BLA), adding significant cost and time burdens [179,180].

### 13.4. Good Manufacturing Practices Requirements for Cell Products

GMP standards form an important foundation for the successful clinical translation of cell-based therapies by ensuring product safety, reproducibility, and compliance with regulatory expectations. Manufacturing these living cell products under GMP conditions requires highly controlled and specialized facilities equipped with cleanrooms, sterile processing equipment, and validated aseptic handling protocols to minimize contamination risks. Essential quality control measures include rigorous testing for sterility, endotoxin presence, mycoplasma contamination, and adventitious agents, alongside standardized procedures for cell isolation, expansion, preservation, and delivery. Comprehensive documentation at every step ensures traceability, reproducibility, and regulatory compliance [24] (Figure 2).

Equally important is a well-trained workforce strictly adhering to standard operating procedures, as human error remains a significant risk factor for GMP compliance breaches. The logistical complexity and high cost of maintaining GMP-compliant facilities and processes, compounded by the biological complexity of living cell therapies, present substantial barriers to wide-scale clinical adoption [24]. Therefore, ongoing translational research and industrial collaborations are focused on developing scalable, automated, and cost-effective GMP-compliant manufacturing platforms. Innovations in automation and flexible manufacturing models are crucial to improving throughput, reducing variability, and expanding patient access while maintaining rigorous quality standards.

## 14. Future Directions

Promising future directions in cell-mediated drug delivery systems include the integration of artificial intelligence (AI) and novel biomaterials to address key challenges in large-scale cell production, viability, functional consistency, and regulatory compliance. AI is applied to optimize cell therapy manufacturing by predicting cell potency, monitoring quality attributes, and automating process control, which reduces batch-to-batch variability and enhances reproducibility [181]. AI-driven analytics can also streamline supply chain logistics and enable real-time, data-driven decision-making throughout the cell therapy life-cycle, supporting compliance with FDA and EMA requirements. In regenerative medicine, AI is used to analyze high-content imaging, predict differentiation outcomes, and design safer, more effective cell products [182]. Advances in biomaterials enable scalable and automated cell expansion while maintaining cell phenotype and function [183]. The convergence of AI, data science, and biomaterial engineering is expected to accelerate the development, standardization, and regulatory approval of cell-based therapies.

Additionally, personalized medicine approaches, such as using a patient’s cells specifically tailored to their disease, offer significant potential in advanced cell-mediated drug delivery systems. Autologous cell therapies can be engineered or loaded with therapeutic agents to address individuals’ disease phenotypes, improving efficacy and safety profiles compared to allogeneic products [184]. They maximize biocompatibility, reduce immune rejection, and enable precise targeting of disease mechanisms. However, large-scale manufacturing of personalized cell products remains a major challenge. Autologous approaches require individualized processing, which complicates standardization, increases costs, and introduces batch-to-batch variability.

## 15. Ethical and Societal Considerations

The development and clinical integration of CMDDS raise several ethical and societal issues that must be addressed alongside technological advancement. These concerns include accessibility, equity, cell sourcing ethics, implications for vulnerable populations, and global disparities in healthcare delivery.

### 15.1. Accessibility and Equity

CMDDSs are inherently complex to manufacture, store, and administer, which can drive high costs for both development and patient care. In many cases, these costs may limit access to well-resourced healthcare systems or privately insured populations [185]. Without targeted policy and reimbursement strategies, wider use of CMDDS could worsen existing gaps in access to dermatologic and regenerative care. Strategies to improve equity may include tiered pricing models, providing subsidies, and facilitating technology transfer to lower-income countries.

### 15.2. Autologous vs. Allogeneic Cell Sourcing Ethics

The choice between autologous and allogeneic cells involves ethical and practical considerations. Autologous therapies, while minimizing immune rejection, require individualized manufacturing that is time-consuming and costly [186]. Allogeneic sources have the capacity for large-scale production and lower per-patient cost, but carry higher risks of immune rejection and potential disease transmission [187]. Donor consent, cell traceability, and transparent oversight of sourcing are essential for maintaining public trust.

### 15.3. Pediatric and Vulnerable Populations

Use of CMDDS in pediatric patients, elderly, and other vulnerable groups requires careful ethical consideration. Long-term safety data are often limited, and the balance between potential benefit and unknown risk may differ significantly compared to adult populations. Informed consent and parental or guardian authorization must be adapted to ensure understanding of both benefits and uncertainties [188].

### 15.4. Global Disparities

Advanced cell-based therapies are more readily available in high-income countries, with significant barriers to access in low- and middle-income settings. Limited infrastructure, lack of trained personnel, and high production costs contribute to this gap [189]. Ethical implementation of CMDDS should consider strategies for global capacity building, such as training partnerships, manufacturing hubs in underserved regions, and international clinical trial inclusion.

### 15.5. Societal Implications and Public Engagement

Public perception of cell-based therapies can influence adoption, policy support, and funding. Misconceptions about safety, origin of cells, or genetic modification can generate resistance, particularly in communities with limited exposure to biomedical technologies [190]. Transparent communication, culturally sensitive education, and stakeholder engagement are essential for fostering informed acceptance.

## 16. Conclusions

CMDDSs represent a rapidly advancing frontier in the management of complex dermatologic diseases. Leveraging the innate homing abilities, immunomodulatory functions, and barrier-crossing properties of living cells, these platforms address key limitations of conventional topical, oral, and injectable therapies, most notably, the challenge of achieving sustained, targeted delivery to diseased skin while minimizing systemic toxicity. Recent progress in nanotechnology, genetic engineering, and exosome-based strategies has broadened the scope of cell-mediated delivery beyond oncology, with promising applications in chronic inflammatory, autoimmune, and regenerative skin conditions.

These case studies, from MSC-exosome therapy for acne and psoriasis to autologous TIL therapy for melanoma, show that CMDDSs are no longer purely theoretical but represent a rapidly maturing translational approach within precision dermatology.

Despite encouraging preclinical and early clinical data, significant hurdles remain for clinical translation. Large-scale cell manufacturing, regulatory compliance, and cost are persistent barriers, and ethical considerations, including equitable access, donor sourcing, and use in vulnerable populations, require careful attention to avoid exacerbating healthcare disparities. The medical literature consistently reinforces the need for validation through multicenter clinical trials to establish safety, efficacy, and cost-effectiveness across diverse patient populations.

If these scientific, regulatory, and ethical challenges are addressed, CMDDSs have the potential to become a cornerstone of precision dermatology. Their capacity for biological targeting and controlled therapeutic release offers the possibility of fundamentally improving outcomes, reducing treatment burden, and advancing personalized care for chronic and refractory skin diseases.

## Figures and Tables

**Table 1:** Comparison of conventional vs. cell-mediated drug delivery systems for dermatological conditions.

Parameter	Topical	Oral	Injectable (Systemic)	Cell-Mediated Drug Delivery System
Target specificity	Low—relies on passive diffusion; limited to superficial lesions	Low—systemic distribution, minimal skin targeting	Medium—higher tissue but not skin-selective	High—uses cell homing to inflamed/malignant skin
Systemic toxicity	Low–Moderate; irritation possible	High—off-target organ toxicity common	Moderate; immune modulation risks	Low—minimizes off-target exposure
Penetration depth	Low; limited by stratum corneum	High (nonspecific)	High (nonspecific)	High (targeted to disease site)
Dosing frequency	High; frequent reapplication	Medium; once-daily to weekly	Low–Medium; every 2–8 weeks	Low; prolonged circulation time and controlled release
Cost	Low ($15–$30/tube)	Medium	High ($12,000–$70,000/yr)	High—variable (early development, potential cost reduction with scale)
Patient adherence	Moderate—frequent dosing reduces compliance	Variable—side effects limit use	Moderate—invasive administration impacts adherence	Potentially high—less frequent dosing, improved tolerability

**Table 2:** In vivo cell types used in CMDDS for dermatologic applications.

Cell Type	Important Properties	Advantages	Limitations	Example Applications
Macrophages	Innate immune cells; phagocytic; home to inflammation via chemokines (CCL2, CXCL12)	Low immunogenicity; cross endothelial barriers; can carry nanoparticles	Potential pro-inflammatory activation; lifespan in vivo varies	Psoriasis, melanoma, chronic wounds
Neutrophils	First-responders to infection/injury; respond to IL-8, LTB4	Rapid recruitment; efficient nanoparticle uptake without loss of migration	Short lifespan; risk of excessive inflammation	Bacterial skin infections, acute inflammatory flares
Dendritic cells	Antigen-presenting cells; initiate adaptive immunity	Potent immune modulation; can deliver antigens for immunotherapy	Complex ex vivo manipulation; limited scalability	Melanoma vaccines, autoimmune modulation
Mesenchymal stem cells	Multipotent stromal cells; strong immunomodulatory secretome	Low immunogenicity; promote regeneration; homing to injury	Donor variability; manufacturing scale limits	Psoriasis, eczema, wound healing
Induced pluripotent stem cells	Reprogrammed somatic cells; differentiate into many lineages	Patient-specific; scalable expansion	Regulatory and safety hurdles; tumorigenicity risk	Vitiligo (melanocyte regeneration), tissue repair
Keratinocytes	Main epidermal cells; can be genetically modified	Local protein delivery; autologous grafting	Limited to accessible lesions; genetic modification required	Gene therapy for genodermatoses, wound healing
Fibroblasts	Dermal ECM-producing cells	Localized sustained release; support tissue repair	Limited migration beyond injection site	Scar reduction, wound healing

## Data Availability

No new data were created or analyzed in this study. Data sharing is not applicable to this article.

## Abbreviations

CMDDS	Cell-Mediated Drug Delivery Systems
DDS	Drug Delivery Systems
MSC	Mesenchymal Stem Cell(s)
iPSC	Induced Pluripotent Stem Cell(s)
EV	Extracellular Vesicle(s)
NET	Neutrophil Extracellular Trap
ROS	Reactive Oxygen Species
DC	Dendritic Cell(s)
APC	Antigen-Presenting Cell
MHC	Major Histocompatibility Complex
Treg	Regulatory T Cell
CTLA-4	Cytotoxic T-Lymphocyte Associated Protein 4
IL	Interleukin (e.g., IL-10, IL-8)
TGF-β	Transforming Growth Factor Beta
PGE2	Prostaglandin E2
IDO	Indoleamine 2,3-Dioxygenase
VCAM-1	Vascular Cell Adhesion Molecule 1
ICAM-1	Intracellular Adhesion Molecule 1
LTB4	Leukotriene B4
FDA	Food and Drug Administration
EMA	European Medicines Agency
